# The Systemic Profile of Soluble Immune Mediators in Patients with Myelodysplastic Syndromes

**DOI:** 10.3390/ijms17071080

**Published:** 2016-07-05

**Authors:** Astrid Olsnes Kittang, Kristoffer Sand, Annette Katharina Brenner, Kristin Paulsen Rye, Øystein Bruserud

**Affiliations:** 1 Department of Clinical Science, University of Bergen, Bergen N-5021, Norway; kristoffer.sand@uib.no (K.S.); annette.brenner@uib.no (A.K.B.); Kristin.Rye@uib.no (K.P.R.); oystein.bruserud@helse-bergen.no (Ø.B.); 2 Division for Hematology, Department of Medicine, Haukeland University Hospital, Bergen N-5021, Norway

**Keywords:** myelodysplastic syndromes, cytokine, chemokine, interleukin, adhesion, MMP

## Abstract

**Introduction:** Myelodysplastic syndromes (MDS) are characterized by bone marrow failure due to disturbed bone marrow maturation. MDS is associated with increased risk of transformation to acute myeloid leukemia (AML) and features of immunological dysregulation. **Materials and methods:** Serum levels of 47 soluble immune mediators were examined in samples derived from 49 MDS patients (35 low-risk and 14 high-risk) and 23 healthy adults. Our patients represent an unselected population-based cohort. The mediators included cytokines, soluble adhesion proteins, matrix metalloproteases, and tissue inhibitors of proteases. Levels were determined using Luminex assays. Patients were classified as low- and high-risk based on the international prognostic scoring system (IPSS) score. **Results:** When comparing the serum levels of single mediators the MDS patients showed a relatively wide variation range for several mediators compared with healthy adults, especially interleukin 6 (IL-6), IL-8/CXCL8, CCL3, and CCL4. The high-risk patients had lower levels of epidermal growth factor (EGF), cluster of differentiation 40 ligand (CD40L), CCL5, CCL11, CXCL5, matrix metalloproteinase 1 (MMP-1), MMP-9, and tissue inhibitor of metalloproteinases 2 (TIMP-2) compared with low-risk patients. Unsupervised hierarchical cluster analysis visualized marked serum mediator profile differences between MDS patients; based on this analysis three patient subsets could be identified. The healthy adults were also included in this analysis and, as expected, they formed their own separate cluster, except for one outlier. Both low- and high-risk patients showed considerable heterogeneity with regard to serum profile, and this heterogeneity seems stable over time (one year follow-up). Finally, very few mediators differed between low- and high-risk patients, but hierarchical clustering based both on all mediators, as well as five selected mediators (EGF, CCL11, TIMP-2, MMP-1, and MMP-9) identified subsets of patients with significantly increased frequency of high-risk disease (χ-square test *p* = 0.0158 and *p* = 0.0148).

## 1. Introduction

Myelodysplastic syndromes (MDS) are a group of bone marrow disorders characterized by dysplastic maturation of bone marrow cells, reduced levels of mature myeloid cells in peripheral blood, and increased risk of transformation to acute myeloid leukemia (AML) [1,2]. MDS can be divided into four different subsets based on the international prognostic scoring system (IPSS), and based on this scoring patients can be classified as low-risk (low and intermediate 1 IPSS classes) and high-risk (intermediate 2 and high risk IPSS classes) [3]. The bone marrow in low-risk MDS is characterized by increased apoptosis, whereas high-risk patients are characterized by accumulation of apoptosis-resistant blasts in bone marrow and, eventually, peripheral blood.

The immune system is postulated to play an important role in the pathophysiology of MDS, at least in a subset of the patients [4,5,6,7]. Low-risk MDS is dominated by proinflammatory cells, while immunosuppressive cells (regulatory T cells and myeloid-derived suppressor cells) [8,9] are more important in high-risk disease where they possibly provide immune evasion for MDS blasts and aid in transformation to AML. The bone marrow environment is important for normal maturation of bone marrow progenitor cells, and signaling through direct cell contact, as well as soluble mediators, are both necessary for correct maturation of progenitor cells [10,11]. Thus, investigation of systemic (i.e., serum or plasma levels) of soluble mediators may reveal information regarding the pathophysiology in MDS, e.g., immunoregulation, as well as regulation of hematopoiesis.

Systemic cytokine profiles (plasma or serum levels) in MDS patients have been investigated in relatively few previous studies [12,13,14]. Kornblau et al. [12] investigated serum levels of cytokines and chemokines for AML and MDS patients. They found major similarities regarding cytokine levels between these two groups, but large differences compared to healthy controls. Feng et al. [13] found increased plasma levels of tumor necrosis factor α (TNFα), interleukin 6 (IL-6), CCL3, interleukin 1 receptor α (IL-1ra), hepatocyte growth factor (HGF), and CCL4 in MDS compared to patients with aplastic anemia (AA) and healthy controls. Epidermal growth factor (EGF) and CXCL5 were then lower in AA as well as MDS compared to controls, whereas only CCL2 was lower in aplastic anemia when compared to MDS. A third study by Pardanani et al. [14] found increased plasma levels of a wide range of cytokines/chemokines in MDS patients compared to healthy controls. Pardanani et al. [14] did not find any significant differences between high- and low-risk MDS, while Feng et al. [13] observed increased CCL4 levels and decreased levels of CCL5, CXCL5, cluster of differentiation 40 ligand (CD40L), vascular endothelial growth factor (VEGF), and EGF in high-risk MDS compared to low-risk disease. This discrepancy calls for further exploration into the cytokine profiles in low- and high-risk MDS. Additionally, these studies did not combine a broad cytokine/chemokine profiling with investigation of biologically different, but functionally related, mediators (i.e., soluble adhesion molecules, proteases, protease inhibitors). For these reasons we have investigated the serum levels of a wide range of biologically diverse, but functionally interacting, soluble mediators (including several cytokines) in a large group of unselected MDS patients.

## 2. Results

### 2.1. The Serum Levels of Single Mediators Show a Wide Variation between Myelodysplastic Syndromes (MDS) Patients

We compared the serum cytokine levels for MDS patients and a group of healthy adults. Our controls were younger than the MDS patients. However, Feng et al. [13] could not detect any age-dependent variation in the systemic levels for any of the cytokines included in our present study, and this has later been confirmed by others for a large number of these cytokines [15]. It is not known whether systemic levels of soluble adhesion molecules, matrix metalloproteases, or tissue inhibitors show any age-dependent variation. For these reasons Table 1 only includes the comparison of systemic cytokine levels for MDS patients and healthy adults, whereas the comparisons for the other mediators are not shown. We used the Mann–Whitney *U* test for these statistical analyses. The patients showed increased levels of several interleukins (IL-5 *p* < 0.001, IL-6 *p* = 0.013, IL-8/CXCL8 *p* < 0.001, IL-13 *p* = 0.001) and chemokines (CCL3 *p* < 0.001, CXCL10 *p* < 0.001), whereas they showed decreased levels for one interleukin (IL-10 *p* < 0.001), two chemokines (CCL5 *p* = 0.010, CXCL5 *p* < 0.001), two immunoregulatory cytokines (interferon γ (IFNγ) *p* < 0.001, CD40L *p* = 0.013), and one growth factor (thrombopoeitin (TPO) *p* < 0.001). Most of these differences were still significant after Bonferroni correction (*p* < 0.0015). Thus, the differences in systemic mediator profiles include a wide range of biologically different cytokines.

It can be seen from Table 1 that a relatively wide variation range was detected for many of the cytokines compared with the healthy controls. The systemic levels of many soluble adhesion molecules, matrix metalloproteases, and tissue inhibitors of matrix proteases also showed a wide variation between the patients.

Most of the significant differences noted above from comparing all MDS patients with controls were also detected when comparing high- and low-risk patients separately with the healthy adults (data not shown). Only four mediators varied differently for high- and low-risk MDS patients; CXCL11 was significantly decreased only for low-risk patients compared to controls (*p* = 0.014) whereas CD40L (*p* = 0.003) and EGF (*p* = 0.008) were decreased only for the high-risk patients. Thus, for the majority of cytokines similar differences were detected when high- and low-risk MDS patients were compared with healthy individuals.

Previous studies have described that age can influence the systemic mediator levels also for patients with myeloid malignancies [16]. However, only CXCL5 (*n* = 49. *r* = 0.343, *p* = 0.017), MMP-3 (*r* = 0.422, *p* = 0.003), TIMP-2 (*r* = 0.362, *p* = 0.011), and TIMP-4 (*r* = 0.506, *p* < 0.001) showed significant correlations with age for our MDS patients. MMP-1, MMP-3, MMP-8, MMP-9, TIMP-3, and P-selectin all showed positive correlation to both absolute neutrophil and lymphocyte counts (data not shown).

We could not detect any correlation between age and the systemic levels of soluble adhesion molecules, MMPs or TIMPs for our healthy controls, and for this reason we used the comparison between MDS patients and healthy controls to illustrate that these mediator levels also showed a considerable variation between patients. The following differences were then observed:

- The patient P-selectin levels were significantly lower than the corresponding levels in the healthy controls (median control levels 83,100 pg/mL, range 43,800–112,000 pg/mL; *p* = 0.001), whereas the patient levels of intercellular adhesion molecule 1 (ICAM-1) (median control level 146,000 pg/mL, range 76,100–329,000 pg/mL, *p* < 0.001) and vascular cell adhesion molecule 1 (VCAM-1) (median control level 652,000 pg/L, range 342,000–1,580,000, *p* < 0.001) were increased. Furthermore, the patient levels did not show any significant correlation with age for any of these soluble adhesion molecules.
- The patient levels were significantly lower than corresponding levels in healthy controls for MMP-2 (median control level 180,000 pg/mL, range 54,400–248,000 pg/mL, *p* < 0.001), MMP-3 (median control level 39,500 pg/mL, range 4600–160,000 pg/mL, *p* < 0.001), MMP-8 (median control level 6380 pg/mL, range 1708–12,100 pg/mL, *p* = 0.039), and MMP-9 (median control level 115,000 pg/mL, range 42,900–333,000 pg/mL, *p* < 0.001). In contrast, the patients showed increased MMP-7 levels compared with the controls (median control level 2060 pg/mL, range 944–8930 pg/mL, *p* < 0.001). Only MMP-3 showed a correlation with age for the MDS patient group.

#### 2.1.1. Serum Mediator Profiles Differ Slightly between High- and Low-Risk MDS Patients

**Figure 1:**
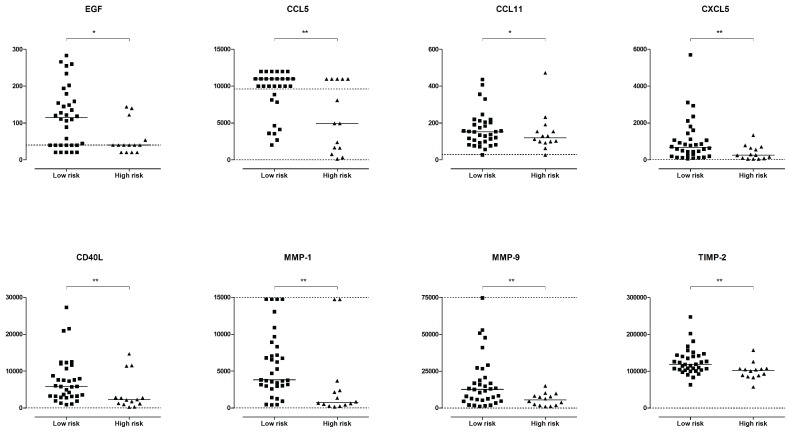
Serum levels of soluble mediators in myelodysplastic syndromes (MDS) patients, a comparison of patients with low-risk (squares) and high-risk (triangles) disease. The figure presents the overall results for those eight mediators showing statistically significant differences between the two groups. The Mann–Whitney *U* test was used for comparison; *p*-values * *p* < 0.05, ** *p* < 0.01. CCL5 was the only mediator with a *p*-value lower than *p* = 0.0013, and considered significant following Bonferroni correction.

MDS is a heterogeneous disease and the inflammatory state seems to differ between low- and high-risk patients [5,17,18]. We, therefore, compared serum levels of cytokines, growth factors, adhesion molecules, and proteases/protease inhibitors between the high- and low-risk MDS patient groups (Table 2). Patients with high-risk MDS had lower serum levels of EGF (Mann–Whitney *U* test, *p* = 0.011), CD40L (*p* = 0.006), CCL5 (*p* = 0.001), CCL11 (*p* = 0.012), and CXCL5 (*p* = 0.004) compared to the low-risk patients (Figure 1). The levels of MMP-1 (*p* = 0.003) and MMP-9 (*p* = 0.010), as well as the inhibitor TIMP-2 (*p* = 0.006), were also lower in high-risk patients (Figure 1). However, CCL5 was the only mediator which retained significance using Bonferroni-corrected *p*-values (*p* < 0.0013). Differences found based on high- and low-risk were confirmed when comparing patients with blast counts below and above five percent, with the exception of CCL11 and MMP-9, which were present in similar levels between the two groups (EGF *p* = 0.002, CD40L *p* = 0.001, CCL5 *p* = 0.004, CCL11 *p* = 0.213, CXCL5 *p* = 0.001, MMP-1 *p* = 0.002, MMP-9 *p* = 0.059, and TIMP-2 *p* = 0.001). Thus, high-risk MDS patients show decreased serum levels of a subset of biologically heterogeneous soluble mediators compared to low-risk patients.

#### 2.1.2. Platelet Counts, Platelet-Derived Mediators and Serum Levels

Platelets contain several mediators that can be released during ex vivo handling of the samples [19]. Platelet counts were available only for 30 of our patients at the time of sampling. We observed significant correlations between platelet counts and serum levels only for CD40L (Spearman correlation, *n* = 30, *r* = 0.689, *p* < 0.001), CCL5 (*r* = 0.569, *p* = 0.001), CXCL5 (*r* = 0.371, *p* = 0.044), VEGF (*r* = 0.610, *p* < 0.001), TPO (*r* = 0.435, *p* = 0.016), and P-selectin (*r* = 0.394, *p* = 0.031) when investigating the whole group which was including both high- and low-risk patients. However, the platelet counts for the low-risk patients were relatively high (median 175 × 10^9^/L, range 26–566) and when these 20 patients were investigated separately we observed significant correlations between peripheral blood platelet counts and CD40L (*r* = 0.751, *p* < 0.001), CCL5 (*r* = 0.652, *p* = 0.002), VEGF (*r* = 0.565, *p* = 0.009), TPO (*r* = 0.516, *p* = 0.020), and P-selectin (*r* = 0.677, *p* = 0.001). No statistically significant correlations were detected when the 10 high-risk patients were examined separately (median 66 × 10^9^/L, range 17–206). These observations suggest that ex vivo platelet release may have a major impact on the serum levels for these mediators.

#### 2.1.3. The Overall Serum Mediator Profile Shows a Wide Variation in MDS Patients and Cannot Distinguish between Low- and High-Risk Patients

**Figure 2:**
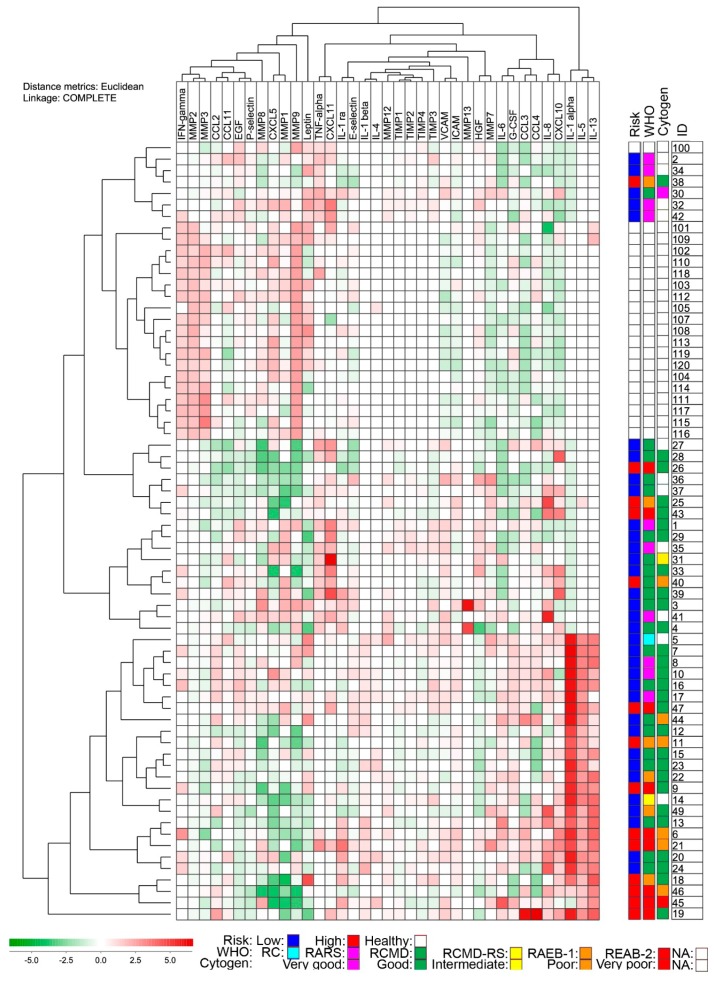
Unsupervised hierarchical clustering of MDS patients and healthy controls. Parameters where 10 or more sample values were above lowest detectable value were included in the clustering analysis. Parameters closely correlated to platelet counts were also excluded from cluster analysis (cluster of differentiation 40 ligand (CD40L), CCL5, vascular endothelial growth factor (VEGF), and thrombopoietin (TPO)). All values were median variance standardized and log(2)-transformed prior to Euclidian distance clustering. Red-to-green gradient (see lower left part of the figure) represents expression levels above or below the median serum levels for each individual parameter, respectively. The classification systems that are used in the right part of the figure with regard to low/high risk myelodysplastic syndromes (MDS) based on international prognostic scoring system (IPSS) (Risk), World Health Organization classification (WHO), and cytogenetic abnormalities (Cytogen) are also explained in the lower part of the figure. Risk: low-risk MDS patients—blue, high-risk MDS patients—red, healthy controls—white. WHO subclasses: refractory cytopenia (RC)—turquoise, refractory anemia with ring sideroblasts (RARS)—magenta, refractory cytopenia with multilineage dysplasia (RCMD)—green, refractory cytopenia with multilineage dysplasia and ring sideroblasts (RCMD-RS)—yellow, refractory anemia with excess blasts-1 (RAEB-1)—orange, RAEB-2—red, data not available (NA)—white. Cytogenetics: very good—magenta, good—green, intermediate—yellow, poor—orange, very poor—red, NA—white.

To further visualize the differences in serum mediator profiles for our MDS patients we did unsupervised hierarchical clustering analyses. The mediators that showed strong correlations between serum levels and platelet counts (CD40L, CCL5, VEGF, and TPO) were left out from these analyses together with those mediators that showed detectable levels in less than 10 samples. We first did an analysis investigating serum levels of 37 soluble mediators from both patients and healthy controls (Figure 2). The individuals then grouped into two major subsets/clusters; all of the healthy individuals were included in the upper cluster and formed a distinct sub-cluster. The upper cluster also included a heterogeneous group of MDS patients. This analysis clearly illustrates that there is a wide variation in systemic cytokine profiles for MDS patients and a minority of patients even shows a profile very similar to healthy individuals. However, an interesting observation is that five out of six patients intermingled between the controls are either RARS or RA with very good prognostic cytogenetics. A major difference between the healthy individuals and patients was caused by compounds in the left major cluster of mediators particularly IFN-γ, MMP-2, MMP-3 (leftmost cluster) and CXCL5, MMP-1, and MMP-9. The lower cluster was mainly defined by higher levels of IL-1α, IL-5, and IL-13 (rightmost cluster). Finally, the frequency of high-risk patients differed between the upper (patients 38, 26, 25, 43, and 40) and lower patient clusters (patients 47, 11, 9, 6, 21, 18, 46, 45, and 19) (four out of 43 in the upper cluster versus nine out of 25 in the lower cluster; χ-square test, *p* = 0.0158). A similar distribution of high-risk patients between, and within, two main clusters was also seen when the clustering analysis only included the patients (data not shown).

**Figure 3:**
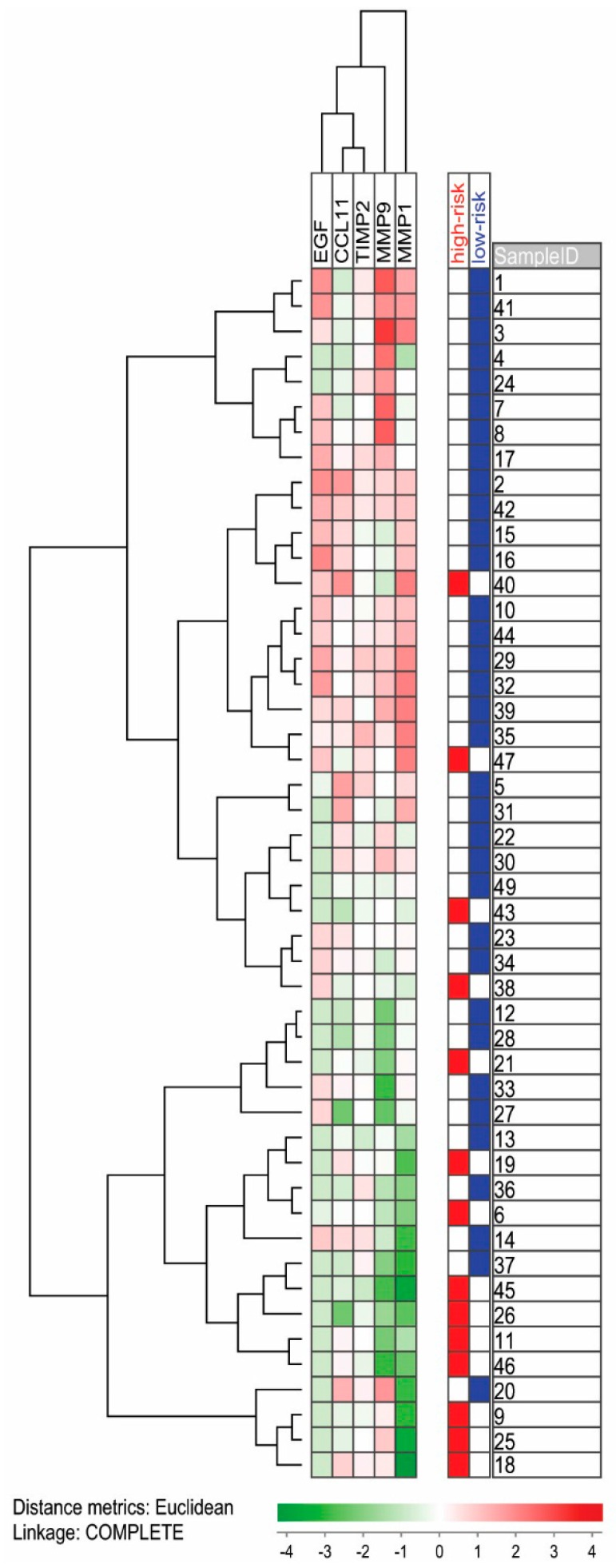
Unsupervised hierarchical clustering analysis of high- and low-risk MDS patients. The analysis was based on five mediators that showed statistically significant differences between the two MDS subsets; we excluded the three mediators CCL5, CXCL5, and CD40L that also showed significant differences but in addition showed significant correlations with platelet counts. All values were median variance standardized and log(2)-transformed prior to Euclidian distance clustering. Red-to-green gradient represents expression levels above or below the median serum levels for each individual parameter, respectively. Each row represents one individual, and low-risk MDS patients are marked with blue and high-risk MDS patients with red.

Finally, we performed a clustering analysis based only on five mediators that showed significant differences between high- and low-risk patients, leaving out CCL5, CXCL5, and CD40L to exclude a possible influence from ex vivo handling of samples (Figure 3). The patients were then separated into two main clusters, and even though the use of this selection of biomarkers were able to group high-risk patients to some extent (four out of 29 in the upper cluster versus 10 out of 19 in the lower cluster; χ-square test, *p* = 0.0148) high-risk patients did not form separate sub-clusters. Taken together with the overall mediator analysis in Figure 2 these last results show that there is a wide variation between MDS patients with regard to their systemic mediator profile, and this variation seems without strong relation to the high/low-risk classification.

#### 2.1.4. Systemic Mediator Profiles Show Only Minor Variation over Time

We could compare the serum levels of soluble mediators at the time of diagnosis and 12 months later for 24 unselected patients (16 low-risk and eight high-risk). Two of the low-risk patients then experienced an increase in bone marrow blasts (1% versus 6.5% and 3% versus 9%, respectively), while the other 14 had stable disease. Two of the high-risk patients transformed to AML before 12 months. One of the low-risk patients and five of the high-risk patients were treated with 5-azacitidine in the 12 months period. When investigating all 24 patients together, the majority of the serum markers were not significantly altered, the exceptions being decreased levels in the 12-month sample of leptin (Wilcoxon’s signed ranks, *n* = 24, *p* = 0.005), MMP-2 (*p* = 0.018), TPO (*p* = 0.026), and ICAM-1 (*p* = 0.040). When the low-risk patients were examined alone decreased leptin levels (*n* = 15, *p* = 0.008) could still be detected and in addition VCAM-1 levels were significantly increased (*p* = 0.007), whereas analysis of the high-risk patients alone showed increased MMP-3 levels (*n* = 8, *p* = 0.012). Thus, the systemic mediator profiles showed only minor variation over time.

## 3. Discussion

The cytokine network is important in orchestrating immune responses, and previous studies suggest that this network is dysregulated in MDS. However, the cytokine network interacts with several other and biologically different immune mediators, e.g., soluble adhesion molecules and the protease system [20,21]. Our study is the first to analyze the systemic (i.e., serum) profile of such a broad range of biologically-heterogeneous soluble mediators in a relatively large group of unselected MDS patients. Previous studies have shown conflicting results regarding systemic cytokine levels in MDS. We observed a wide variation of the soluble mediator profile in serum from MDS patients and this variation showed no strong association with the established prognostic classification of MDS patients.

Our healthy controls were younger than the MDS patients, but two previous studies failed to show any age-dependent differences between healthy individuals for a majority of the cytokines that they examined [13,15]. One of these studies compared the levels of all cytokines included in our present study in different age groups [13]. To the best of our knowledge it is not known whether systemic levels of soluble adhesion molecules, MMPs, and TIMPs correlate with age. We, therefore, solely compared serum cytokine levels for the MDS patients and our healthy controls in Table 1.

The levels of several soluble adhesion molecules and MMPs differed between our patients and healthy controls and these adhesion molecules/MMPs did not show any correlation with age neither for our patients (MMP-3 being the only exception), nor for the healthy controls. However, despite this, we would emphasize that these results have to be interpreted with great care.

Only serum samples were available for our study, and platelets may then become activated and release soluble mediators ex vivo during sample preparation with activation of the coagulation system [19,22]. A significant correlation between serum mediator levels and peripheral blood platelet counts would then be expected if this ex vivo release caused a significant contribution to the serum levels, and this was observed only for a minority of the mediators examined in our study. On the other hand, it would not be surprising if the true systemic in vivo levels of platelet-associated mediator levels were correlated with the peripheral blood platelet counts, and this is also supported by studies of plasma samples [19]. Whether one should leave out or include serum levels of platelet-derived mediators in analyses of serum mediator profiles is, thus, controversial. For the clustering analysis in the present study we either left out those mediators showing very strong correlations with platelet counts (Figure 2) or we left out all showing significant correlations (Figure 3).

MMPs and their inhibitors should be regarded as immunoregulatory mediators and show several interactions with the chemokine/cytokine system [21]. Very few previous studies have investigated MMP expression in MDS [23,24,25,26], and these studies examined the intracellular expression or the release during in vitro culture of bone marrow mononuclear cells. In contrast to the high release of MMP-2 by bone marrow mononuclear cells described by Ries et al. [23] and Travaglino et al. [25] we observed decreased levels of MMP-2 in serum from MDS patients, and our present results thereby suggest that MMP-2 release by non-myeloid cells and/or MMP-2 degradation/absorption are more important for the systemic levels than the release by immature myeloid cells. Our observation that MMP-1/3/9 serum levels correlate with neutrophil and lymphocyte peripheral blood counts are also consistent with the hypothesis that the serum MMP levels reflect release by various cells. Former observations regarding MMP-9 are heterogeneous; Messingerová et al. [26] observed increased MMP-9 levels in MDS patients with del(5q), and these levels normalized after treatment with lenalidomide, Travaglino et al. [25] observed normal levels in MDS and decreased levels in patients with AML, while Ries et al. [23] found decreased MMP-9 compared to healthy controls. These observations are consistent with our present observations that there is a wide distribution range for MDS patients with regard to MMP-9 levels. Travaglino et al. [25] observed inverse correlations between the frequency of bone marrow blasts and release of MMP-2, as well as MMP-9 by myeloid cells. Observations by Iwata et al. [24] also suggested reduced potential for MMP-9 induction in monocytes from MDS patients with excess of blasts. These previous observations may explain our present observation of lower MMP-2/3/9 serum levels in high-risk patients. Finally, MMP-7 serum levels were increased in MDS patients and showed no correlation to peripheral blood cell counts. MMP-7 is involved in cleavage of Fas ligands [27], and the increased levels may, thus, affect immune surveillance or regulation of apoptosis in MDS patients.

The results from the two previous studies of soluble adhesion molecules in MDS are conflicting; Südhoff et al. [28] found no difference between MDS patients and healthy controls, whereas Passam et al. [29] reported increased systemic levels of both VCAM-1 and ICAM-1 and this increase was most pronounced in high-risk disease. Both studies described significant correlations between these adhesion molecule levels and peripheral blood monocyte counts, and Passam et al. [29]. in addition observed an association between high levels and adverse prognosis. We also detected increased serum levels of VCAM-1 and ICAM-1 in MDS, and higher median levels in high-risk disease, but we could not detect any association with prognosis based on IPSS scores. Of note, the overlap between low- and high-risk groups for these adhesion molecules was greater than between MDS patients and controls, and a lack of significant association is, thus, not likely to be caused by smaller sample size.

We observed increased levels of CXCL8/IL-8 and CXCL10 in MDS compared to controls in our cohort of unselected patients, and these chemokines clustered together in Figure 2. CXCL8/IL-8 is (i) a chemoattractant, primarily for neutrophils; and (ii) a pro-angiogenic factor [30]. CXCL8 has previously been described as the quantitatively-dominant pro-angiogenic factor in AML [31], found to be increased in MDS and AML [32]. Further, release of CXCL8/IL8 has been found to be increased when AML blasts are co-cultured with osteoblasts [33]. CXCL8/IL-8 binds the receptor CXCR2 and the inhibition of this axis is proposed as a potentially specific therapeutic target in MDS and AML [32]. Increased CXCL10 levels have also been detected in previous studies and has been suggested as an independent poor prognostic factor [14]; our present results describing no difference between low- and high-risk patients defined by the IPSS score is also consistent with the hypothesis that CXCL10 levels have an independent prognostic impact. CXCL10 binds to the CXCR3 chemokine receptor and acts as (i) a chemoattractant, particularly for Th1 and Tc1 effector lymphocytes [34,35,36]; (ii) an angioregulatory mediator [37]; and (iii) a regulator of hematopoiesis [38]. The previously-described increased expression of CXCR3 mRNA both in peripheral blood and bone marrow mononuclear cells further support a role of CXCL10 in the pathogenesis of MDS [39].

## 4. Materials and Methods

### 4.1. Patients

The study was approved by the Regional Ethics Committee (REK vest; REK number 3.2008.409), and samples were collected after written informed consent. Serum samples were collected at the time of diagnosis for 49 patients (35 low-risk and 14 high-risk) (Supplementary Table S1), and a second sample was collected one year later for 24 of them (16 low-risk, eight high-risk). The male/female ratio for the MDS patients were 3.1:1 and the median age was 74 years (54–93). Serum samples from 23 healthy individuals were also collected (male/female ratio 0.6:1, median age 38 years (26–68)). Haukeland University Hospital (Bergen, Norway) is the primary hospital for a defined geographic area and our patient population represents an unselected/consecutive group of MDS patients from this single institution. Patients were risk-stratified according to the international prognostic scoring system (IPSS) [3], and patients with classification as low and intermediate-1 IPSS were grouped together as low-risk, while intermediate-2 and high IPSS classification are referred to as high-risk. Three patients who scored as intermediate-1 IPSS, were included in the high-risk group as patient one (patient 18, see Supplementary Table S1) showed clinical progression with increasing BM blast counts (to 9%) and increasing cytopenias following sampling, the second (patient 25) showed a rapid increase in bone marrow blast counts (to 18%) within 10 weeks after sampling and the third patient (patient 40) had a major discrepancy to international prognostic scorin system revised (IPSS-R) (score 4.0—intermediate risk) and increasing bone marrow blast counts (to 6%) within 15 weeks of sampling.

### 4.2. Sample Preparation and Analysis

Peripheral blood samples were left to coagulate at room temperature for 60 min before centrifugation (1300× *g* for 10 min) and subsequent serum collection. Samples were stored at −80 °C until the levels of 47 soluble mediators were determined by Luminex analyses (R&D Systems, Minneapolis, MN, USA): (i) the eleven interleukins IL-1α, IL-1β, IL-2, IL-4, IL-5, IL-6, IL-10, IL-12p70(IL-12), IL-13, IL-17, and interleukin 1 receptor antagonist (IL-1ra); (ii) the nine chemokines CCL-2/3/4/5/11, CXCL8/IL-8 and CXCL-5/10/11; (iii) the eight growth factors granulocyte colony-stimulating factor (G-CSF), granulocyte macrophage colony-stimulating factor (GM-CSF), VEGF, TPO, EGF, fibroblast growth factor basic (FGF-B), HGF, and leptin; (iv) the three immunomodulatory cytokines IFN-γ, CD40L, and TNF-α; (v) the four soluble adhesion molecules P-selectin, E-selectin, ICAM-1, and VCAM-1; and (vi) the eight matrix metalloproteinases (MMP)-1/2/3/7/8/9/12/13 together with four tissue inhibitors of metalloproteinases (TIMP)-1/2/3/4. Six of these mediators (FGF-B, GM-CSF, IL-2, IL-12, IL-17, and MMP-13) were undetectable for more than 90% of samples, and were excluded from the analyses. Samples from 48 MDS patients (34 low-risk and 14 high-risk) and 20 healthy individuals were analyzed for cytokine/chemokine and growth factor levels, whereas samples from all 49 patients and 23 controls were analyzed for protease and adhesion molecule levels; TIMP levels were not analyzed for the controls.

### 4.3. Statistical and Bioinformatical Analyses

Statistical analyses were performed using IBM SPSS (IBM Corp., Armonk, NY, USA) and Graph Pad Prism (GraphPad Software, La Jolla, CA, USA, used for analysis of paired samples). Mann–Whitney *U* test was used when comparing non-parametric values. Paired samples were analyzed using Wilcoxon’s signed ranks test. Correlation analysis for nonparametric values was performed using Spearman’s test. *p*-Values below 0.05 were considered significant. Bioinformatical analyses were performed using J-Express (MolMine AS, Bergen, Norway) [40]. For unsupervised hierarchical clustering all values were median variance standardized and log(2)-transformed. Complete linkage was used as linkage method, and for distance measured, the Euclidian correlation was used.

## 5. Conclusions

To conclude, we investigated the systemic/serum profile of 47 biologically different soluble immune mediators (i.e., cytokines/chemokines, soluble adhesion molecules, proteases and their inhibitors) for a cohort of unselected MDS patients, and we describe: (i) differences between MDS patients and healthy individuals for several of these biologically different mediators but, at the same time, a considerable heterogeneity within the MDS cohort with regard to their serum profile of soluble immune mediators; (ii) this wide variation is seen both for patients with low- and high-risk disease, (iii) low- and high-risk patients differ only for a minority of these mediators; and (iv) this mediator profile is relatively stable over time in the individual patient, and this seems to be true even for patients with disease progression requiring treatment with hypomethylating agents. Based on our present results we suggest that the possible prognostic or predictive impact of the systemic profile of soluble immune mediators should be further investigated in future clinical studies.

## Figures and Tables

**Table 1:** Serum levels of soluble mediators; a comparison between myelodysplastic syndromes (MDS) patients and healthy individuals.

Mediator	MDS Patients	Healthy Individuals	*p*-Value
*Cytokines*			
IL-1ra	761 (182–10,311)	852 (503–1700)	**<0.001**
IL-1α	5.04 (bd–241)	n/a	n/a
IL-1β *	bd (bd–6.99)	n/a	n/a
IL-4	bd (bd–51.4)	bd (bd–29.5)	ns
IL-5	4.17 (bd–16.3)	bd (bd–1.57)	**<0.001**
IL-6 *	5.85 (bd–88.7)	3.47 (bd–5.23)	0.013
IL-10 *	bd (bd–22.8)	3.87 (2.62–5.33)	**<0.001**
IL-13	bd (bd–362)	bd (bd–66.7)	**0.001**
*Chemokines*			
CCL2 *	276 (bd–1570)	268 (146–632)	ns
CCL3	113 (bd–6370)	55.2 (bd–137)	**<0.001**
CCL4 *	66.0 (bd–6320)	64.7 (14.0–150)	ns
CCL5	ad (134–ad)	ad (5060–ad)	0.010
CCL11	136 (bd–283)	114 (30.4–500)	ns
CXCL5	566 (bd–5700)	1 590 (425–4200)	**<0.001**
CXCL8 *	41.0 (8.10–1220)	14.5 (1.69–37.8)	**<0.001**
CXCL10 *	59.0 (7.00–590)	17.1 (9.16–57.6)	**<0.001**
CXCL11 *	40.5 (bd–2690)	bd (bd–167)	ns
*Immunomodulatory cytokines*			
TNFα	2.01 (bd–24.9)	3.17 (bd–17.8)	ns
IFNγ *	bd (bd–6.97)	2.94 (bd–3.57)	**<0.001**
CD40L *	3940 (264–27,300)	7560 (4470–22,600)	0.013
*Growth factors*			
EGF *	73.4 (bd–283)	80.1 (40.2–222)	ns
VEGF *	86.5 (bd–275)	84.2 (10.4–264)	ns
TPO	467 (110–2058)	779 (174–1057)	**<0.001**
HGF	275 (31.0–1605)	385 (175–545)	ns
G-CSF *	33.9 (bd–140)	25.6 (bd–71.3)	ns
Leptin	4750 (622–148,000)	10,500 (1400–46,600)	ns
*Adhesion molecules; serum levels in MDS patients*			
E-Selectin	28,400 (6770–107,000)	ICAM-1	272,000 (84,100–589,000)
P-Selectin	56,200 (18,100–126,000)	VCAM-1	1,220,000 (552,000–4,540,000)
*Matrix metalloproteinases and inhibitors; serum levels in MDS patients*			
MMP-1	3570 (184–14,800)	MMP-12	8.50 (bd–38.3)
MMP-2	45,800 (36,000–62,500)	TIMP-1	166,000 (91,000–257,000)
MMP-3	12,500 (2720–17,800)	TIMP-2	108,000 (57,400–248,000)
MMP-7	4810 (993–31,100)	TIMP-3	27,400 (bd–77,400)
MMP-8	4260 (350–35,600)	TIMP-4	2080 (1110–4170)
MMP-9	8110 (1040–74,700)		

All mediators are given as pg/mL. The Mann–Whitney *U* test is used for comparison between the two groups for each mediator, and the corresponding *p*-values still significant after Bonferroni correction are given in bold (*p*-values < 0.0015). Fibroblast growth factor basic (FGF-B), granulocyte macrophage colony-stimulating factor (GM-CSF), interleukin 2 (IL-2), IL-12, IL-17, and matrix metalloproteinase 13 (MMP-13) are not included in the table as all or most serum levels of these mediators were undetectable. Serum levels of IL-1α, IL1-β, MMP-12, and tissue inhibitors of metalloproteinases 1-4 (TIMP1-4) were only investigated for MDS patients. ad; above detection level; bd, below detection level; n/a, not available; ns, not significant; tumor necrosis factor (TNF); interferon (IFN), cluster of differentiation 40 ligand (CD40L); epidermal growth factor (EGF); vascular endothelial growth factor (VEGF); thrombopoietin (TPO); hepatocyte growth factor (HGF); granulocyte colony-stimulating factor (G-CSF); intercellular adhesion molecule 1 (ICAM-1), vascular cell adhesion molecule 1 (VCAM-1). * A previous study could not detect any age-dependent differences for these mediators except for CD40L that showed higher levels in elderly individuals, while G-CSF and CCL2 showed lower levels in elderly individuals [15].

**Table 2:** Serum levels of soluble mediators; a comparison between low- and high-risk MDS patients.

Mediator	Low-Risk MDS	High-Risk MDS	*p*-Value
*Cytokines*			
IL-1ra	665 (318–4610)	1032 (182–10,300)	ns
IL-1α	2.75 (bd–241)	23.9 (bd–203)	ns
IL-1β	bd (bd–6.99)	0.87 (bd–4.04)	ns
IL-4	bd (bd–46.0)	bd (bd–51.4)	ns
IL-5	bd (bd–16.3)	4.55 (bd–16.1)	ns
IL-6	5.55 (bd–39.2)	5.85 (bd–88.7)	ns
IL-13	bd (bd–292)	103 (bd–362)	ns
*Chemokines*			
CCL2	276 (bd–1570)	268 (146–632)	ns
CCL3	113 (bd–6370)	55.2 (bd–137)	ns
CCL4	66.0 (bd–6320)	64.7 (14.0–150)	ns
CCL5	ad (2010–ad)	ad (134–ad)	**0.001**
CCL11	136 (bd–283)	114 (30.4–500)	0.012
CXCL5	566 (bd–5700)	1590 (425–4200)	0.004
CXCL8	41.0 (8.1–1220)	14.5 (1.69–37.8)	ns
CXCL10	59.0 (7.0–590)	17.1 (9.16–57.6)	ns
CXCL11	40.5 (bd–2690)	bd (bd–167)	ns
*Immunomodulatory cytokines*			
TNFα	4.02 (bd–17.8)	bd (bd–24.9)	ns
IFNγ	bd (bd–3.35)	bd (bd–6.97)	ns
CD40L	5830 (918–27,300)	2260 (264–14,700)	0.006
*Growth factors*			
EGF	115 (bd–283)	bd (bd–144)	0.011
VEGF	91.1 (17.1–191)	69.1 (bd–275)	ns
TPO	497 (110–1770)	439 (154–2060)	ns
HGF	290 (31.0–1610)	273 (91.7–634)	ns
G-CSF	32.4 (bd–89.0)	38.7 (bd–140)	ns
Leptin	4560 (622–54,700)	4750 (786–148,000)	ns
*Adhesion molecules*			
E-Selectin	28,600 (6770–107,000)	24,700 (8310–59,700)	ns
P-Selectin	57,200 (18,100–126,000)	54,800 (21,400–94,800)	ns
ICAM-1	239,000 (84,100–503,000)	303,000 (118,000–589,000)	ns
VCAM-1	1,180,000 (553,000–4,540,000)	1,380,000 (870,000–2,770,000)	ns
*Matrix metalloproteinases and inhibitors*			
MMP-1	3820 (425–ad)	777 (184–ad)	0.003
MMP-2	45,900 (37,300–62,500)	44,700 (36,000–62,200)	ns
MMP-3	12,500 (5240–ad)	12,100 (2720–ad)	ns
MMP-7	4710 (993–28,400)	6100 (1640–31,100)	ns
MMP-8	4260 (438–35,600)	4100 (350–24,100)	ns
MMP-9	12,500 (1070–74,700)	5510 (1040–15,100)	0.010
MMP-12	8.20 (bd–38.3)	8.50 (bd–16.2)	ns
TIMP-1	167,000 (98,400–257,000)	135,000 (91,000–245,000)	ns
TIMP-2	119,000 (62,700–248,000)	102,000 (57,400–158,000)	0.006
TIMP-3	34,300 (bd–77,400)	9180 (bd–77,300)	ns
TIMP-4	2120 (1110–3500)	1880 (1280–4170)	ns

All mediators are given as pg/mL. The Mann–Whitney *U* test is used for comparison between low-risk and high-risk MDS patients for each mediator and the corresponding *p*-values still significant after Bonferroni correction are given in bold (*p*-values < 0.0013). FGF-basic. IL-2, IL-12, IL-17, and MMP-13 are not included in the table as all or most serum levels of these mediators were undetectable. ad, above detection level; bd, below detection level; ns, not significant.

## Supplementary Materials

Supplementary materials can be found at http://www.mdpi.com/1422-0067/17/7/1080/s1.

## References

[1] Corey S.J., Minden M.D., Barber D.L., Kantarjian H., Wang J.C., Schimmer A.D. (2007). Myelodysplastic syndromes: The complexity of stem-cell diseases. Nat. Rev. Cancer.

[2] Nimer S.D. (2008). Myelodysplastic syndromes. Blood.

[3] Greenberg P., Cox C., LeBeau M.M., Fenaux P., Morel P., Sanz G., Sanz M., Vallespi T., Hamblin T., Oscier D. (1997). International scoring system for evaluating prognosis in myelodysplastic syndromes. Blood.

[4] Sloand E.M., Mainwaring L., Fuhrer M., Ramkissoon S., Risitano A.M., Keyvanafar K., Lu J., Basu A., Barrett A.J., Young N.S. (2005). Preferential suppression of trisomy 8 compared with normal hematopoietic cell growth by autologous lymphocytes in patients with trisomy 8 myelodysplastic syndrome. Blood.

[5] Kordasti S.Y., Afzali B., Lim Z., Ingram W., Hayden J., Barber L., Matthews K., Chelliah R., Guinn B., Lombardi G. (2009). IL-17-producing CD^4+^ T cells, pro-inflammatory cytokines and apoptosis are increased in low risk myelodysplastic syndrome. Br. J. Haematol..

[6] Zheng Z., Qianqiao Z., Qi H., Feng X., Chunkang C., Xiao L. (2010). In vitro deprivation of CD^8+^CD^57+^ T cells promotes the malignant growth of bone marrow colony cells in patients with lower-risk myelodysplastic syndrome. Exp. Hematol..

[7] Chen X., Eksioglu E.A., Zhou J., Zhang L., Djeu J., Fortenbery N., Epling-Burnette P., van Bijnen S., Dolstra H., Cannon J. (2013). Induction of myelodysplasia by myeloid-derived suppressor cells. J. Clin. Investig..

[8] Kordasti S.Y., Ingram W., Hayden J., Darling D., Barber L., Afzali B., Lombardi G., Wlodarski M.W., Maciejewski J.P., Farzaneh F. (2007). CD4^+^CD25^high^ Foxp3^+^ regulatory T cells in myelodysplastic syndrome (MDS). Blood.

[9] Kittang A.O., Kordasti S., Sand K.E., Costantini B., Kramer A.M., Perezabellan P., Seidl T., Rye K.P., Hagen K.M., Kulasekararaj A. (2016). Expansion of myeloid derived suppressor cells correlates with number of t regulatory cells and disease progression in myelodysplastic syndrome. Oncoimmunology.

[10] Verfaillie C.M. (1998). Adhesion receptors as regulators of the hematopoietic process. Blood.

[11] Kornblau S.M., Cohen A.C., Soper D., Huang Y.W., Cesano A. (2014). Age-related changes of healthy bone marrow cell signaling in response to growth factors provide insight into low risk mds. Cytom. B Clin. Cytom..

[12] Kornblau S.M., McCue D., Singh N., Chen W., Estrov Z., Coombes K.R. (2010). Recurrent expression signatures of cytokines and chemokines are present and are independently prognostic in acute myelogenous leukemia and myelodysplasia. Blood.

[13] Feng X., Scheinberg P., Wu C.O., Samsel L., Nunez O., Prince C., Ganetzky R.D., McCoy J.P., Maciejewski J.P., Young N.S. (2011). Cytokine signature profiles in acquired aplastic anemia and myelodysplastic syndromes. Haematologica.

[14] Pardanani A., Finke C., Lasho T.L., Al-Kali A., Begna K.H., Hanson C.A., Tefferi A. (2012). IPSS-independent prognostic value of plasma CXCL10, IL-7 AND IL-6 levels in myelodysplastic syndromes. Leukemia.

[15] Kim H.O., Kim H.S., Youn J.C., Shin E.C., Park S. (2011). Serum cytokine profiles in healthy young and elderly population assessed using multiplexed bead-based immunoassays. J. Transl. Med..

[16] Olsnes A.M., Motorin D., Ryningen A., Zaritskey A.Y., Bruserud O. (2006). T lymphocyte chemotactic chemokines in acute myelogenous leukemia (AML): Local release by native human aml blasts and systemic levels of CXCL10 (IP-10), CCL5 (rantes) and CCL17 (TARC). Cancer Immunol. Immunother..

[17] Koike M., Ishiyama T., Tomoyasu S., Tsuruoka N. (1995). Spontaneous cytokine overproduction by peripheral blood mononuclear cells from patients with myelodysplastic syndromes and aplastic anemia. Leuk. Res..

[18] Wu L., Li X., Chang C., Ying S., He Q., Pu Q. (2008). Deviation of type I and type II T cells and its negative effect on hematopoiesis in myelodysplastic syndrome. Int. J. Lab. Hematol..

[19] Tvedt T.H., Rye K.P., Reikvam H., Brenner A.K., Bruserud O. (2015). The importance of sample collection when using single cytokine levels and systemic cytokine profiles as biomarkers—A comparative study of serum versus plasma samples. J. Immunol. Methods.

[20] Bruserud O., Halstensen A., Peen E., Solberg C.O. (1996). Serum levels of adhesion molecules and cytokines in patients with acute leukaemia. Leuk. Lymphoma.

[21] Hatfield K.J., Reikvam H., Bruserud O. (2010). The crosstalk between the matrix metalloprotease system and the chemokine network in acute myeloid leukemia. Curr. Med. Chem..

[22] Bruserud O. (2013). Bidirectional crosstalk between platelets and monocytes initiated by toll-like receptor: An important step in the early defense against fungal infections?. Platelets.

[23] Ries C., Loher F., Zang C., Ismair M.G., Petrides P.E. (1999). Matrix metalloproteinase production by bone marrow mononuclear cells from normal individuals and patients with acute and chronic myeloid leukemia or myelodysplastic syndromes. Clin. Cancer Res..

[24] Iwata M., Pillai M., Ramakrishnan A., Hackman R.C., Deeg H.J., Opdenakker G., Torok-Storb B. (2007). Reduced expression of inducible gelatinase B/matrix metalloproteinase-9 in monocytes from patients with myelodysplastic syndrome: Correlation of inducible levels with the percentage of cytogenetically marked cells and with marrow cellularity. Blood.

[25] Travaglino E., Benatti C., Malcovati L., Della Porta M.G., Galli A., Bonetti E., Rosti V., Cazzola M., Invernizzi R. (2008). Biological and clinical relevance of matrix metalloproteinases 2 and 9 in acute myeloid leukaemias and myelodysplastic syndromes. Eur. J. Haematol..

[26] Messingerova L., Jonasova A., Barancik M., Polekova L., Seres M., Gibalova L., Breier A., Sulova Z. (2015). Lenalidomide treatment induced the normalization of marker protein levels in blood plasma of patients with 5q-myelodysplastic syndrome. Gen. Physiol. Biophys..

[27] Mitsiades N., Yu W.H., Poulaki V., Tsokos M., Stamenkovic I. (2001). Matrix metalloproteinase-7-mediated cleavage of fas ligand protects tumor cells from chemotherapeutic drug cytotoxicity. Cancer Res..

[28] Sudhoff T., Germing U., Aul C. (2002). Levels of circulating endothelial adhesion molecules in patients with myelodysplastic syndromes. Int. J. Oncol..

[29] Passam F.H., Tsirakis G., Boula A., Fragou A., Consolas I., Alegakis A., Kyriakou D.S., Alexandrakis M.G. (2004). Levels of soluble forms of ICAM and VCAM in patients with myelodysplastic syndromes and their prognostic significance. Clin. Lab. Haematol..

[30] Waugh D.J., Wilson C. (2008). The interleukin-8 pathway in cancer. Clin. Cancer Res..

[31] Glenjen N., Hovland R., Wergeland L., Wendelbo O., Ernst P., Bruserud O. (2003). The angioregulatory phenotype of native human acute myelogenous leukemia cells: Influence of karyotype, Flt3 abnormalities and differentiation status. Eur. J. Haematol..

[32] Schinke C., Giricz O., Li W., Shastri A., Gordon S., Barreyro L., Bhagat T., Bhattacharyya S., Ramachandra N., Bartenstein M. (2015). IL8-CXCR2 pathway inhibition as a therapeutic strategy against mds and aml stem cells. Blood.

[33] Bruserud O., Ryningen A., Wergeland L., Glenjen N.I., Gjertsen B.T. (2004). Osteoblasts increase proliferation and release of pro-angiogenic interleukin 8 by native human acute myelogenous leukemia blasts. Haematologica.

[34] Appay V., van Lier R.A., Sallusto F., Roederer M. (2008). Phenotype and function of human T lymphocyte subsets: Consensus and issues. Cytom. A.

[35] Bonecchi R., Bianchi G., Bordignon P.P., D’Ambrosio D., Lang R., Borsatti A., Sozzani S., Allavena P., Gray P.A., Mantovani A. (1998). Differential expression of chemokine receptors and chemotactic responsiveness of type 1T helper cells (Th1s) and Th2s. J. Exp. Med..

[36] Sallusto F., Lenig D., Mackay C.R., Lanzavecchia A. (1998). Flexible programs of chemokine receptor expression on human polarized T helper 1 and 2 lymphocytes. J. Exp. Med..

[37] Strieter R.M., Kunkel S.L., Arenberg D.A., Burdick M.D., Polverini P.J. (1995). Interferon γ-inducible Protein 10 (IP-10), a member of the C-X-C chemokine family, is an inhibitor of angiogenesis. Biochem. Biophys. Res. Commun..

[38] Liu M., Guo S., Stiles J.K. (2011). The emerging role of CXCL10 in cancer (review). Oncol. Lett..

[39] Ge M., Zheng Y., Li X., Lu S., Li H., Chen F., Chen D., Shao Y., Shi J., Feng S. (2013). Differential expression profile of Th1/Th17/Th2-related chemokines and their receptors in patients with acquired bone marrow failure syndromes. Hum. Immunol..

[40] Stavrum A.K., Petersen K., Jonassen I., Dysvik B. (2008). Analysis of gene-expression data using J-Express. Curr. Protoc. Bioinform..

