# Supplementary material for: The Systemic Profile of Soluble Immune Mediators in Patients with Myelodysplastic Syndromes
Source: Int J Mol Sci. 2016 Jul 5;17(7):1080. doi: 10.3390/ijms17071080 (PMC4964456; doi:10.3390/ijms17071080)
Supplement: Supplementary file 1 [file ijms-17-01080-s001.pdf]

Supplementary Materials: The Systemic Profile of Soluble Immune Mediators in Patients with Myelodysplastic Syndromes

Astrid Olsnes Kittang, Kristoffer Sand, Annette Katharina Brenner, Kristin Paulsen Rye and Øystein Bruserud

Table S1. Patients included in the study, with patient and disease characteristics.

ID [1]	Gender [2]	Age [3]	Diagnosis [4]	WHO-Class [5]	Cytopenia(s) [6]	Cytogenetics [7]
Low and intermediate-1 interational prognostic scoring system (IPSS)						
1	M	69	2007	RARS	A	46 XY
2	M	81	2008	RARS	A	n/a
3	M	71	2010	RCMD	A	46 XY
4	M	74	2008	RCMD	A, T	46 XY
5	M	70	2009	RC	A, N, T	n/a
7	F	86	2012	RCMD	A	46 XX
8	M	76	2000	RARS	A, T	46 XY
10	F	70	2011	RARS	A	46 XX
12	M	78	2008	RCMD	A, T	46 XY
13	M	80	2011	RCMD	A, N, T	46 XY
14	M	93	2011	RCMD-RS	A	n/a
15	M	72	2011	RCMD	N, T	46XY
16	M	60	2011	RCMD	A	46 XY
17	M	82	2011	RARS	A	46 XY
20	M	81	2012	RCMD	A, T	46 XY
22	M	82	2012	RAEB-1	A	46 XY
23	F	78	2012	RCMD	A, N, T	46 XX
24	M	91	2012	RCMD	A, N, T	46 XY
27	F	85	2008	RCMD	A, N	n/a
28	M	74	2008	RCMD	A, T	46 XY
29	M	83	2009	RCMD	A	46 XY
30	M	81	2009	RCMD	A	45 X
31	M	85	2007	RCMD	A, T	45 XY, del(11)(q23) *
32	F	85	2000	RARS	A	n/a
33	M	55	2009	RCMD	A, N	46 XY
34	F	84	2000	RARS	A	n/a
35	F	89	2005	RARS	A	n/a
36	M	63	2002	RCMD	A, T	n/a
37	M	88	2009	RCMD	A, N, T	n/a
39	M	59	2009	RCMD	A	46 XY
41	M	85	2005	RARS	A	n/a
42	F	75	2006	RARS	A	n/a
44	M	56	2012	RCMD	N, T	47 XY, +1
48	M	76	2000	RARS	A, T	46 XY
49	M	83	2013	RAEB-1	A, N, T	46 XY

Table S1. *Cont.*

ID [1]	Gender [2]	Age [3]	Diagnosis [4]	WHO-Class [5]	Cytopenia(s) [6]	Cytogenetics [7]
				Intermediate-2 and high IPSS		
6	F	57	2010	RAEB-2	A, N, T	46 XX, del(5)(q15q33)/ 46 XX, idem, add(17)(p13), −22, +mar. (Ratio 9/11)
9	F	68	2009	RAEB-2	A, N, T	46 XX, del(12)(p11)
11	M	67	2010	RAEB-1	A, N, T	43 XY complex
18	F	73	2012	RAEB-1	N, T	46 XX
19	M	54	2012	RAEB-2	A, N, T	46 XY
21	M	79	2012	RAEB-2	A	47 XY, +11, +8, −9
25	F	75	2010	RAEB-1	A, N, T	46 XX
26	M	66	2011	RAEB-2	A, N, T	46 XY
38	M	69	2008	RAEB-1	A, N, T	46 XY, del(12)(p12)/46 XY. (Ratio 3/17)
40	M	68	2010	RAEB-1	N	46 XY, t(3;6)(q26;q25) *
43	M	66	2010	RAEB-2	A, N, T	46 XY
45	M	61	2010	RAEB-2	A, T	44 XY/45 XY complex
46	M	71	2012	RAEB-2	A, N, T	47 XY, +8
47	M	66	2012	RAEB-2	A, T	46 XY

[1] Patient identification number; [2] Gender male (M) and female (F); [3] Age at sampling; [4] Year diagnosed with myelodysplastic syndromes (MDS); [5] World Health Organization (WHO) 2008 classification. Refractory anemia with ringed sideroblasts (RARS); refractory cytopenia (RC); refractory cytopenia with multilineage dysplasia (RCMD); Refractory anemia with excess blasts-1 (RAEB-1); RAEB-2; [6] Anemia (A); neutropenia (N); thrombocytopenia (T); [7] Cytogenetics based on karyotyping. Not acquired (n/a), * cytogenetics based on samples from 2011.
